# The POR rs10954732 polymorphism decreases susceptibility to hepatocellular carcinoma and hepsin as a prognostic biomarker correlated with immune infiltration based on proteomics

**DOI:** 10.1186/s12967-022-03282-1

**Published:** 2022-02-14

**Authors:** Yan Fang, Hongming Yang, Guiming Hu, Jiakun Lu, Jun Zhou, Na Gao, Yuhan Gu, Cunzhen Zhang, Jinhuan Qiu, Yuanyuan Guo, Yunfei Zhang, Qiang Wen, Hailing Qiao

**Affiliations:** 1 Institute of Clinical Pharmacology, Zhengzhou University, Zhengzhou, 450052 China; 2 Affiliated People’s Hospital of Zhengzhou University, Zhengzhou, China; 3 Affiliated Cancer Hospital of Zhengzhou University, Zhengzhou, China

**Keywords:** Hepatocellular carcinoma, POR, Polymorphisms, HPN, Proteomics

## Abstract

The effect of the cytochrome P450 oxidoreductase (POR) rs10954732 (*G* > *A*) polymorphism on hepatocellular carcinoma (HCC) susceptibility is unknown. Here we found that *A* allele carriers showed a 69% decrease in susceptibility to HCC with overall survival (OS) prolonged to 199%, accompanied by lower activity for cytochrome P450 2E1. A total of 222 differentially expressed proteins were mainly enriched in neutrophil and T cell activation and involved in the immune and inflammatory responses, constituting the altered immune tumor microenvironment related with A allele by proteomics analysis. Hepsin (HPN) showed significant down-regulation in HCC and up-regulation in A allele carriers. A lower HPN level was associated with increased susceptibility to HCC and a worse prognosis. Moreover, HPN is a potential independent prognostic biomarker for HCC and is strongly associated with clinicopathological features, tumor-infiltrating status of immune cells both in our discovery cohort and database surveys. Our findings provide a new potential mechanism by which HPN may play an important role in the susceptibility of rs10954732 *A* allele carriers to HCC and their prognosis through tumor immune infiltration, thus offering potential insights for future studies on tumor immunotherapy.

**Supplementary Information:**

The online version contains supplementary material available at 10.1186/s12967-022-03282-1.

## Introduction

Hepatocellular carcinoma (HCC) accounts for approximately 70%-90% of primary liver cancer and is the seventh most common cancer and the third leading cause of cancer-related deaths worldwide [1]. Due to its characteristics of poor clinical prognosis, high mortality rates, and frequent recurrence, identifying early risk factors or potential biomarkers that identify high-risk individuals for early detection, diagnosis, and treatment to improve HCC outcomes is of paramount importance. Although much effort has been directed toward identifying multifactorial risk factors for HCC, the predictive and/or prognostic value of currently identified risk factors for HCC is limited, and a more reliable biological risk marker has yet to be identified [2].

It is now well established that a complex combination of genetic and environmental factors contributes to HCC hepatocarcinogenesis, including polymorphisms of the *IL-23R* gene^3^, ErbB4 [4], p53^5^, TNF-α [6] and XPC codon [7], hepatitis virus infection, carcinogenic exposure, and so on. During the past several years, increasing evidence indicated that genes that encode metabolic enzymes can serve as tumor susceptibility genes and/or prognostic signatures, in part through their roles in activation of carcinogens; these genes include cytochrome P450 2E1 (CYP2E1) [8, 9], CYP2D6 [10], GSTP1 [11], NAT2 and GSTM1 [12]. Our previous systematic studies on 10 major CYP enzymes responsible for hepatic drug metabolism showed significant changes in CYP polymorphism incidence [10] and enzyme activity [13–15] in HCC patients, some of which were confirmed as related to increased susceptibility to hepatofibrosis and HCC [16, 17], especially for increased activity of CYP2D6 and CYP2E1, decreased activity of CYP2C8, and decreased frequency of the CYP2D6*2 allele.

Cytochrome P450 oxidoreductase (POR), is the unique electron donor for all microsomal CYP enzymes. This enzyme directly participates in the metabolism of exogenous chemicals and drugs [18, 19]. *POR* gene knock-out or liver-specific deletion has demonstrated to be lethal in the embryonic stage and causes a severe disruption of hepatic drug metabolism. In addition to the widespread expression of POR in multiple normal and tumor tissues [20, 21], the *POR* gene is highly polymorphic, making it possible that *POR* variations contribute to cancer risk, either by changes in the metabolic activation of environmental carcinogens or by affecting the electron transfer process and metabolic activity of CYP enzymes [22]. Therefore, it is reasonable to study in detail *POR* polymorphisms and HCC susceptibility.

A growing number of studies have identified *POR* polymorphisms as risk factors associated with prognosis in multiple cancers, including breast cancer [23] and bladder cancer [21]. However, only isolated reports have reported that one *POR* variant, *A503V*, is associated with HCC risk and prognosis, and the detailed mechanism by which *POR* polymorphisms confer HCC susceptibility and progression has not been addressed. The reason is partly because phenotypic changes associated with diseases, especially tumors, resulting from genotypic changes are a complex process involving many proteins, making it difficult to study potential mechanisms in detail.

Thus, this study aimed to characterize the association between *POR* polymorphisms and HCC susceptibility and prognosis in 85 normal subjects and 100 HCC patients. Mass spectrometry (MS)-based proteomics emerged as the preferred strategy and provides a sensitive and accurate method for large-scale proteomic analyses [24]. We used this approach with 34 normal livers and peritumors of 60 HCC samples to elucidate changes in the tumor microenvironment (TME) related to the *POR* *rs10459732* (*G* > *A*) polymorphism and to identify mechanisms for HCC susceptibility and prognosis associated with this polymorphism. Among the altered TME, hepsin (HPN), which has been associated with the growth and progression of of various cancers [25–28], showed the most significant alteration in our discovery cohort. In contrast to the significant down-regulation in the HCC group, expression of HPN was firstly discovered with higher expression in HCC bearing *POR* *rs10459732 A* allele mutation compared with wild-type, which may partly account for decreased HCC susceptibility underlying the *rs10954732 A* allele. Furthermore, apart from evaluation in in our discovery cohort, expression of HPN, its correlation with prognosis in HCC, and the status of different tumor-infiltrating immune cells based on expression of specific markers were also comprehensively validated by experimental research and database surveys from ONCOMINE, Kaplan–Meier plotter, Gene Expression Profiling Interactive Analysis (GEPIA), and the Tumor Immune Estimation Resource (TIMER) databases.

## Methods

### Study design

**Fig. 1:**
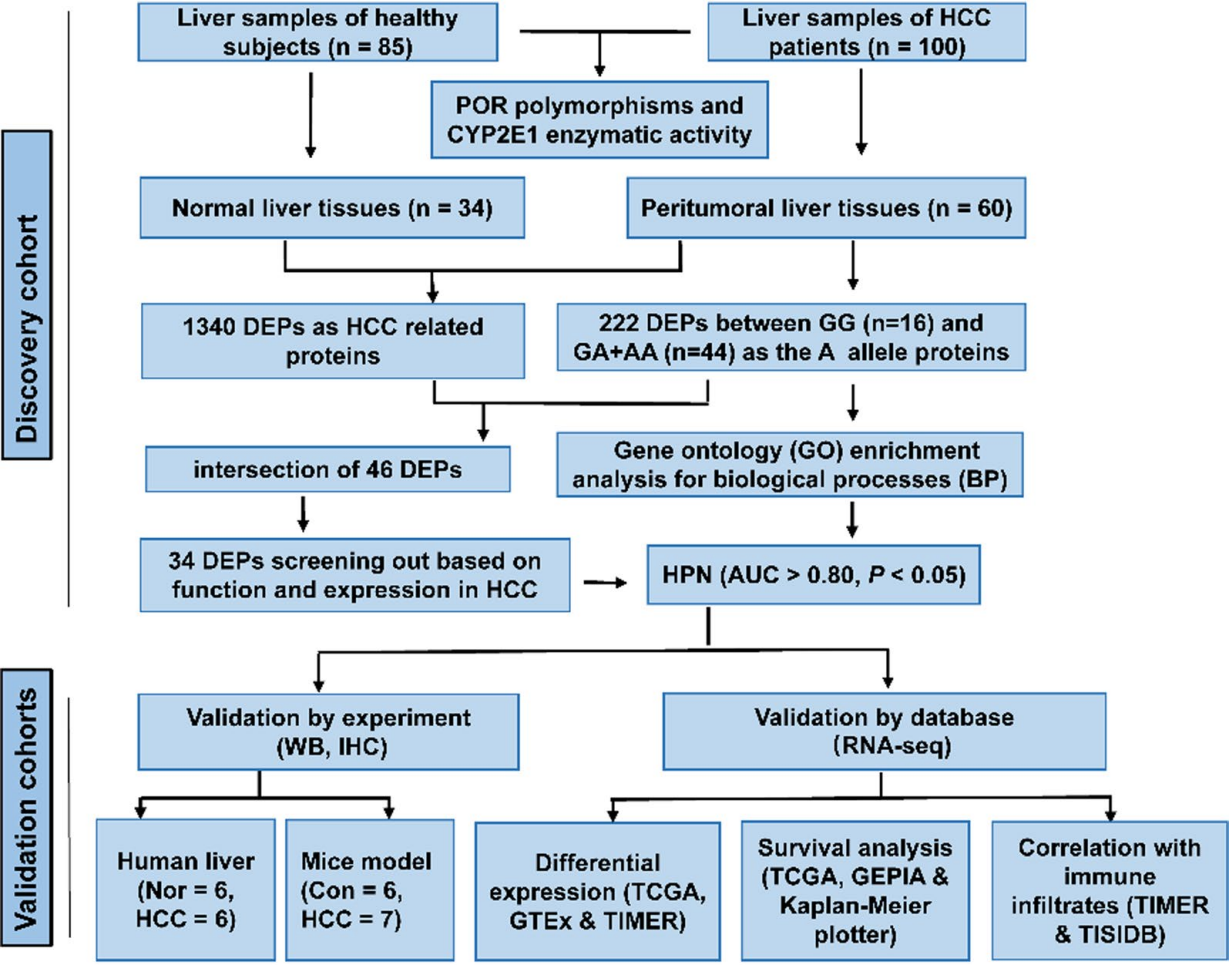
An overview of the experimental design workflow. Illustration of the potential mechanism underlying the role of *POR rs10954732* (*G* > *A*) polymorphisms in decreased HCC susceptibility and increased OS of HCC using label-free quantitative proteomic analysis. POR cytochrome P450 oxidoreductase, *HCC* hepatocellular carcinoma, *OS* overall survival time. A carriers refers to rs10954732 *GA*+*AA* genotypes; *GG* refers to wild type. *DEPs* differentially expressed proteins, *HPN* hepsin, *AUC* area under the ROC curves. Go enrichment for BP, assigning Gene Ontology (GO) terms to proteins, specifying involvement in biological processes (BP). Nor means normal group based on human liver samples. Con means control group based on HCC mouse model. *RNA-seq* RNA sequencing, *HCC* hepatocellular carcinoma, *ELISA* enzyme-linked immunosorbent assay, *WB* western blot, *IHC* immunohistochemistry

The study consisted of a discovery cohort and some validation cohorts. A schematic diagram of our workflow is presented in Fig. 1. The discovery cohort is composed of liver samples from 85 healthy subjects and 100 HCC patients undergoing hepatic surgery (Additional file 1: Table S1). The validation cohorts consisted of experimental research studies and database surveys. Experimental research was conducted with human and mouse liver tissues and paraffin specimens. Database surveys were performed from GTEx, TCGA, TIMER, GEPIA, Kaplan–Meier plotter and TISIDB databases.

### Human liver samples

Normal liver samples were obtained from patients undergoing hepatic surgery and usually diagnosed with hepatic haemangioma, and only tissue specimens with normal liver function and histological appearance were collected. Peritumor liver samples were obtained from HCC patients without receiving tumor radiotherapy, chemotherapy or targeted drug therapy before surgery; tissue specimens were taken 2 cm away from the tumor tissues. Liver specimens were stored in liquid nitrogen within 30 min after resection. Well-documented demographic information including gender, age and laboratory tests were obtained from an inpatient case record [13]. All liver samples were collected from the First Affiliated Hospital of Zhengzhou University, the Affiliated People's Hospital of Zhengzhou University and the Affiliated Cancer Hospital of Zhengzhou University. This study was carried out in accordance with the Declaration of Helsinki and approved by the Medical Ethics Committee of Zhengzhou University. Written informed consent was provided by all donors.

### Genotypes of POR and Activity of CYP2E1

The genomic DNA purification kit (Beijing ComWin Biotech Co., Ltd., China) was used to isolate genomic DNA from liver tissues. Four polymorphisms for POR with frequencies more than 1% in the Chinese population based on consideration of linkage disequilibrium (LD) analysis were genotyped by polymerase chain reaction (PCR) sequencing according to our previous study [29]. Human liver microsomes (HLMs) were prepared by differential centrifugation and enzyme activity of CYP2E1 in HLMs was determined by measuring the rate of chlorzoxazone 6-hydroxylation by high-performance liquid chromatography with eight chlorzoxazone concentrations (7.8–1000 µM) as reported previously [30]. Chlorzoxazone and 6-OH-chlorzoxazone were purchased from the National Institute for the Food and Drug Control (China) and Toronto Research Chemicals, Inc. (Canada), respectively. The incubation system contained HLMs (0.3 mg/mL protein), 100 mM phosphate buffer (pH 7.4) and serial concentrations of substrate. The reaction was initiated with 1 mM NADPH with an optimal incubation time of 30 min. One mL of ethyl acetate was added to terminate the reactions and the metabolite of 6-OH-chlorzoxazone was determined by HPLC–UV. Examination on linear range, precision, relative recovery, and stability were all determined. The Michaelis–Menten affinity constant (K_m_) and maximum reaction velocity (V_max_) values was determined according to nonlinear regression analysis by GraphPad Prism 8.03 software. The intrinsic clearance CL_int_ was estimated by the Michaelis–Menten parameter estimates (CL_int_ = V_max_ / K_m_).

### Proteomics and data analysis

The proteomic analysis based on normal livers and peritumor tissue from HCC patients was conducted using liquid chromatography-mass spectrometry-tandem mass spectrometry (Q-Exactive HF LC–MS/MS) by the State Key Laboratory of Proteomics, Beijing Proteome Research Center, (Beijing, China). Raw data from mass spectrometry were processed against the human UniProt protein sequence database (version 20,140,922, 20,193 sequences). Protein expression is shown as intensity-based absolute protein quantification (iBAQ) based on peak intensity. Proteomic data was normalized by log2 transformation. Proteins that were undetectable in more than 50% of samples were excluded, and missing values for each protein were replaced with half of the minimum. Further detailed methods on proteomic processing and analysis are according to a previous report [31].

### Functional enrichment analysis

The R/Bioconductor package limma v.3.24.15 34 [32] was used to identify differentially expressed proteins (DEPs) between 34 normal livers and peritumor tissues of 60 HCC patients as HCC related proteins, or between GG genotype and GA + AA genotypes in peritumor tissues of 60 HCC patients as A allele-related proteins. Proteins with both differences greater than 1.2 and *P* < 0.05 were considered significant. Potential biological functions of DEPs were identified by Gene ontology (GO) analysis for Enrich GO function in the R Package “clusterProfiler”. GO analysis was according to the threshold of *P* < 0.05 and q < 0.05.

### Allograft transplantation model

Male BALB/c mice (18–22 g) were purchased from Beijing Vital River Laboratory Animal Technology Co. Ltd. Mice were first anesthetized with 300 mg/kg chloral hydrate by intraperitoneal injection. Each mouse was implanted in the left liver lobe with 10 μL of an H22 cell suspension (1.5 × 10^6^ cells/mL;10 μL for each mouse) or 10 μL of serum-free RPMI-1640 medium (sham operation group) by a subcapsular intrahepatic injection. The mice were kept in a Specific Pathogen Free (SPF) warm incubator until they had recovered from the anesthesia and then returned to the animal room.

### Validation of HPN expression in HCC

HPN expression in the livers of 6 normal subjects and 6 peritumor tissues of HCC patients was determined by western blotting (WB) with primary anti-HPN antibody (ab189246, optimal dilutions at 1:1500). Expression of CD68, CD163 and IL-10 in liver samples for the HCC mouse model was determined by immunohistochemistry (IHC). Validation of HPN expression in various cancers including HCC was obtained by exploring the GTEx, TCGA and TIMER databases.

### Validation of prognostic value of HPN in HCC

The correlation of HPN mRNA level with clinical prognosis was evaluated by analyzing the TCGA database, the Kaplan–Meier plotter database and the GEPIA database. Subsequently, the correlation of HPN with the tumor immune cell infiltration level was assessed by exploring the TIMER platform and TISIDB website. The prognostic value of HPN expression in predicting prognosis based on immune cells was determined by analyzing the Kaplan–Meier plotter database.

### Statistical analysis

Statistical analyses were carried out using SPSS version 24.0 software (SPSS Inc. Chicago, IL, USA). Data are expressed as mean ± SE. The Wilcoxon Mann–Whitney test was used for pairwise comparison between two independent groups. Receiver operating characteristic (ROC) curves were constructed to evaluate the models' predictive values in terms of sensitivity, specificity, and respective areas under the curves (AUCs). *P* values are 2-sided and a *P* value of less than 0.05 is defined as statistically significant.

## Results

### *rs10954732* (*G* > *A*) polymorphisms and HCC susceptibility

**Fig. 2:**
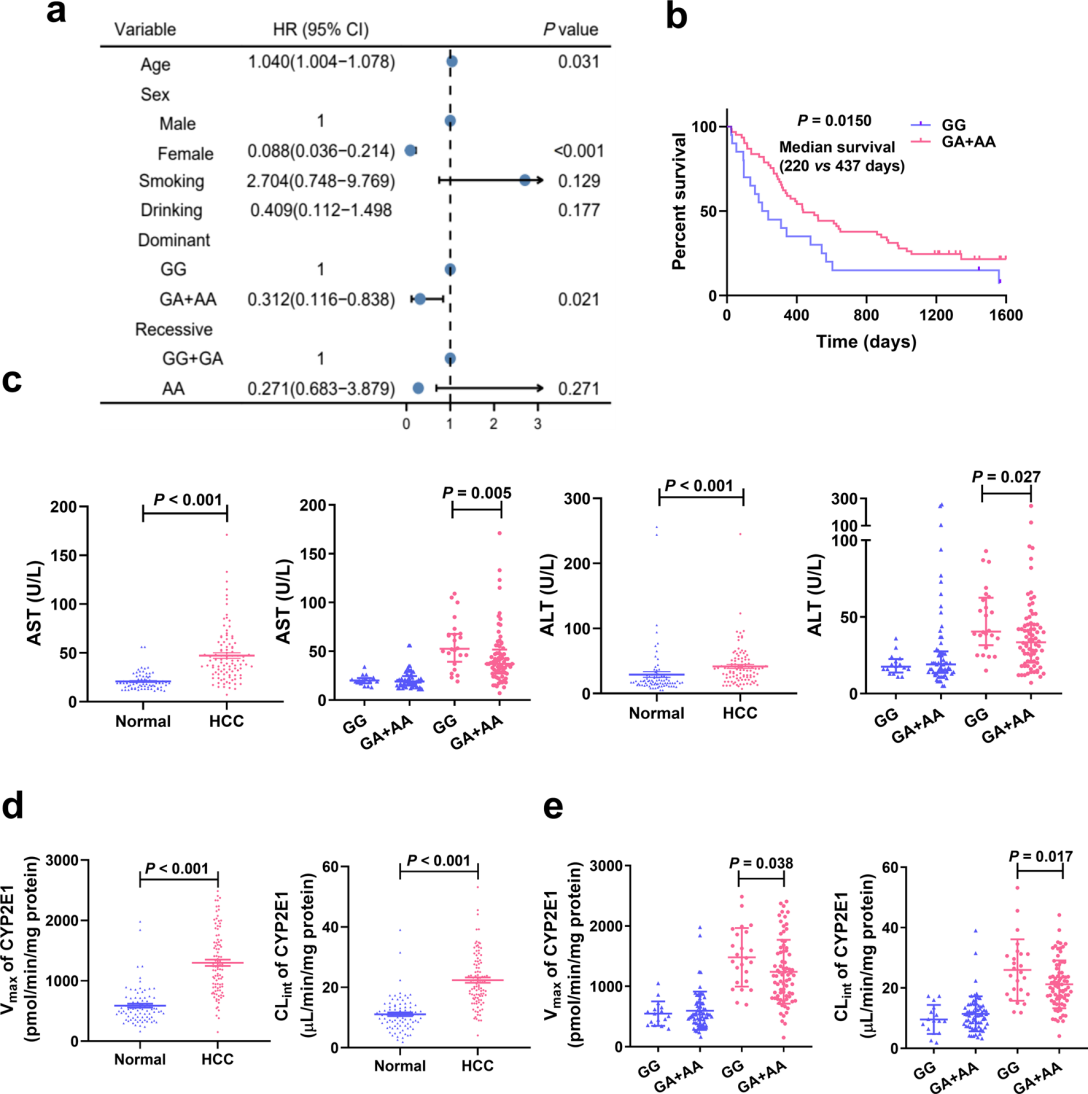
*POR rs10954732* polymorphisms related to decreased HCC susceptibility and increased OS of HCC patients as well as enzyme activity for CYP2E1 in healthy subjects and HCC patients. **a** Univariate logistic regression analysis of the association between *rs10954732* polymorphisms and the risk of HCC. **b** Kaplan–Meier curve for overall survival (OS) of HCC patients between the *GG* genotype and *GA* + *AA* genotypes in HCC patients. Biochemical data (**c**) and CYP2E1 activity (**d**) in subjects between *GG* genotype and *A* allele carriers in healthy subjects and HCC patients. *A* allele carriers, *GA* + *AA* genotypes. Normal, healthy subjects. HCC, hepatocellular carcinoma. *AST* transaminase, *ALT* alanine aminotransferase, *V*_*max*_ maximum reaction velocity, *CL*_*int*_ intrinsic clearance. Data are expressed as mean ± SEM, *P* value was calculated by Mann–Whitney U test

A Hardy–Weinberg equilibrium test indicated that the genotype distribution of the four SNPS (*rs10954732*, *rs2286822*, *rs1135612* and *rs1057868*) for POR included in the study were in Hardy–Weinberg equilibrium (Additional file 1: Table S2). The *rs10954732* (*G* > *A*) polymorphism was associated with significantly lowered susceptibility to HCC, demonstrated by a 68.8% decreased risk of HCC in individuals with *GA* + *AA* genotype (*A* allele carriers) ((Odds Ratio (OR) = 0.312, 95% confidence interval (CI), 0.116–0.838, *P* = 0.021)) in comparison with *GG* wild-type (Fig. 2a), suggesting that the rs10954732 A allele carrier is a protective factor for HCC. For the remaining polymorphisms (*rs2286822*, *rs1135612* and *rs1057868*) no significant relationship was detected between these three polymorphisms and HCC risks (Additional file 1: Table S2). The protective factor of the *rs10954732* *A* allele for HCC was further substantiated by examining the prognosis of HCC patients. As depicted in the Kaplan–Meier survival curve, patients with the *A* allele were more likely to have longer overall survival (OS) compared with the *GG* wild-type (median OS, 437 vs 220 days, *P* = 0.0150, Fig. 2b). Taken together, these results suggest an important role for the *rs10954732* *GA* + *AA* genotype in decreased HCC susceptibility and longer OS.

Moreover, serum biochemical measurements revealed higher levels of aspartate transaminase (AST) and alanine aminotransferase (ALT) in patients than in the normal group, while lower levels in patients with the *A* allele when compared with individuals carrying the *GG* genotype (Fig. 2c), results which further corroborated the protective role of the A allele. To explore the underlying mechanism, the effect of the *G* > *A* mutation on activity of cytochrome P450 2E1 (CYP2E1), an enzyme involved in hepatocarcinogenesis, was evaluated. In line with AST and ALT, significantly increased CYP2E1 activity was found in the HCC group (Fig. 2d), whereas decreased CYP2E1 activity was found in *A* allele carriers (Fig. 2e). These results suggest that the decreased HCC susceptibility associated with the* rs10954732* *A* allele might be related to the lowered metabolic activity of CYP2E1.

### Proteomic mechanisms underlying *A* allele-related susceptibility to HCC

**Fig. 3:**
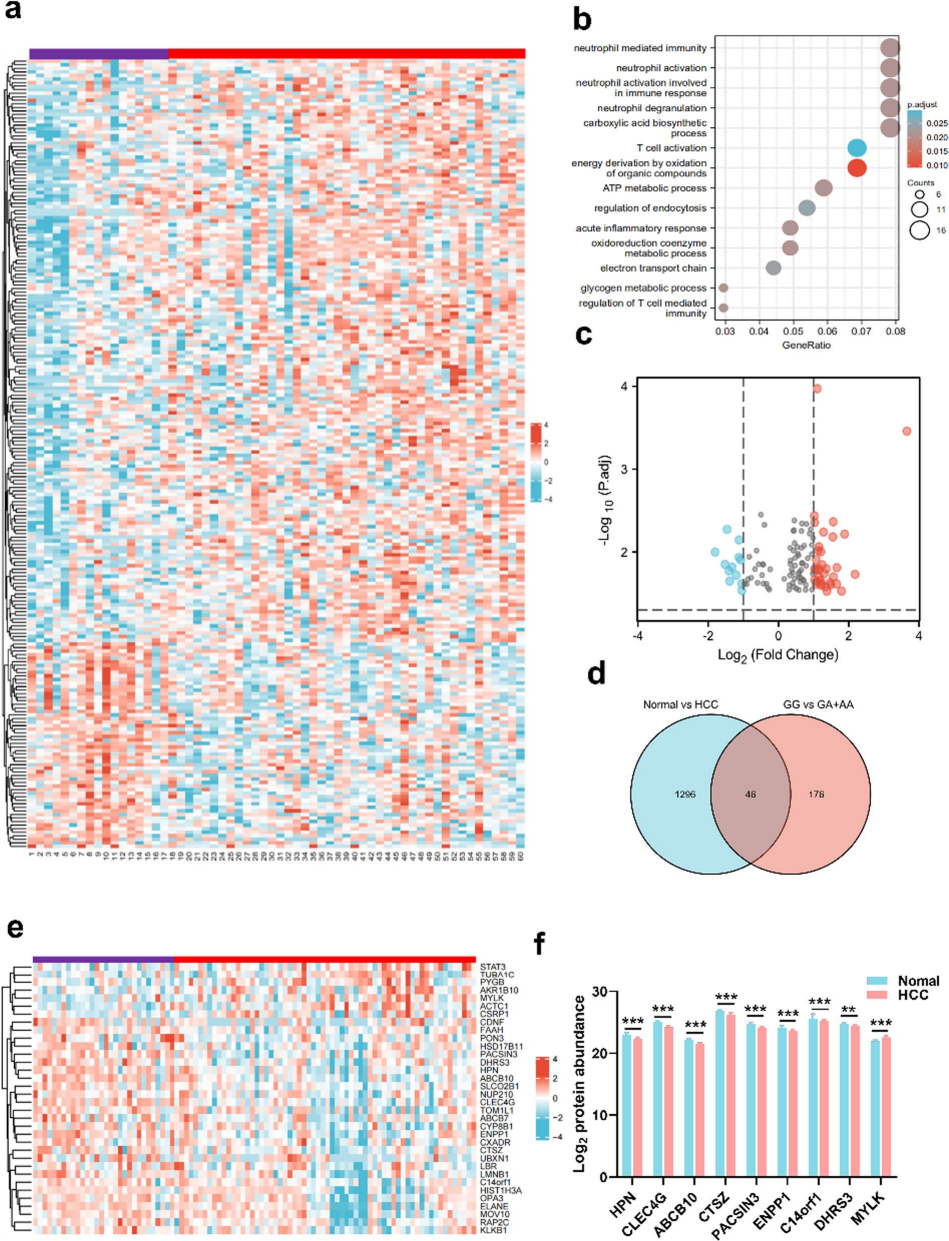
Proteomic mechanisms for *POR* *rs10954732* polymorphisms to confer decreased susceptibility to HCC. **a** A total of 222 DEPs related to *rs10954732* *A* allele compared with the *GG* genotype in HCC patients as shown in a heat map. GO enrichment analysis (**b)** and Volcano plot (**c**) for 222 DEPs with dysregulation related with *A* allele carriers. **d** Wayne figure shows 46 DEPs intersections between 222 *A* allele-related DEPs and 1342 HCC-related DEPs. **e** Heat map of 34 DEPs with protein changes showing the opposite direction in the HCC group and *A* allele carriers. **f** Expression profiles of the top eight significantly up-regulated and one down-regulated DEPs in peritumoral tissues of HCC. Data are shown as mean ± SEM, **P < 0.01, ***P < 0.001 vs Normal group by Mann–Whitney U test. *DEPs* differentially expressed proteins, *HCC* hepatocellular carcinoma

To explore the mechanism underlying the reduced HCC susceptibility associated with the rs10954732 polymorphism, proteomic analysis of liver tissues from 16 *GG* genotypes and 44 *GA* + *AA* genotypes derived from the 60 HCC patients was performed to identify *rs10954732*-related DEPs. There were 222 DEPs identified with the heat-map-based screening criteria of |log2-fold change (FC)|≥ 0.20 and *P* value < 0.05 (Fig. 3a). Subsequently, 117 statistically significant DEPs including 86 up-regulated and 31 down-regulated proteins in the *GA* + *AA* genotypes were selected after adjusting for *P* < 0.05, as shown in the volcano plot (Fig. 3b).

Further functional analysis of the DEPs by gene ontology (GO) enrichment analysis shows that dysregulated proteins were significantly enriched in immune, inflammation and metabolism, as demonstrated by neutrophil and T cell activation, immune and inflammatory response, energy derivation, ATP, oxidoreduction coenzyme and glycogen metabolic processes, regulation of endocytosis, and electron transport chain (Fig. 3c). These results suggest an altered tumor-extrinsic microenvironment in *GA* + *AA* genotypes by regulation of the immune response, inflammatory response and altered metabolic reprogramming, which in part provides an underlying mechanism by which the A allele confers a lower risk of HCC.

In addition to *A* allele-related proteins, a further proteomic analysis with 60 HCC patients and 34 normal subjects identified 1342 DEPs, which are considered as HCC-related proteins. The intersection between the 222 *A* allele-related DEPs and the 1342 HCC-related DEPs identified 46 proteins (Fig. 3d). Changing trends of expression for these 46 *A* allele-related DEPs in HCC compared with normal group and in the *rs10954732* *GA* + *AA* genotype compared with *GG* genotype was next investigated. Surprisingly, an opposite trend was observed in HCC patients when grouped by *rs10954732* mutation. Specifically, we found that 27/36 (75.0%) proteins up-regulated in the A allele carriers showed significant down-regulation, and 7/10 (70.0%) proteins down-regulated in individuals carrying *A* allele were up-regulated in HCC patients (Fig. 3e). The opposite direction for protein changes in the HCC group and A allele carriers once again confirmed the protective role of the *A* allele mutation.

### Hepsin may play an underlying protective role with the A allele in HCC

We speculate that the above 34 proteins with opposite expression trends in HCC patients when grouped by the rs10954732 mutation might be involved in lowered susceptibility to HCC. Therefore, we focused the 34 A allele-related DEPs with regard to already-known cancer-related proteins based on a thorough literature review, and receiver operating characteristic (ROC) curves was constructed. Nine out of 34 proteins showed the most notable changes in expression in HCC patients with a high AUC (> 0.70) (Fig. 3f), indicating high power to distinguish HCC patients from healthy subjects. The significant top DEPs included 8 down-regulated proteins (HPN, CLEC4G, ABCB10, CTSZ, PACSIN3, ENPP1, C14orf1 and DHRS3) and one up-regulated protein (MYLK). Among them, hepsin (HPN) showed the largest AUC at 0.802.

**Fig. 4:**
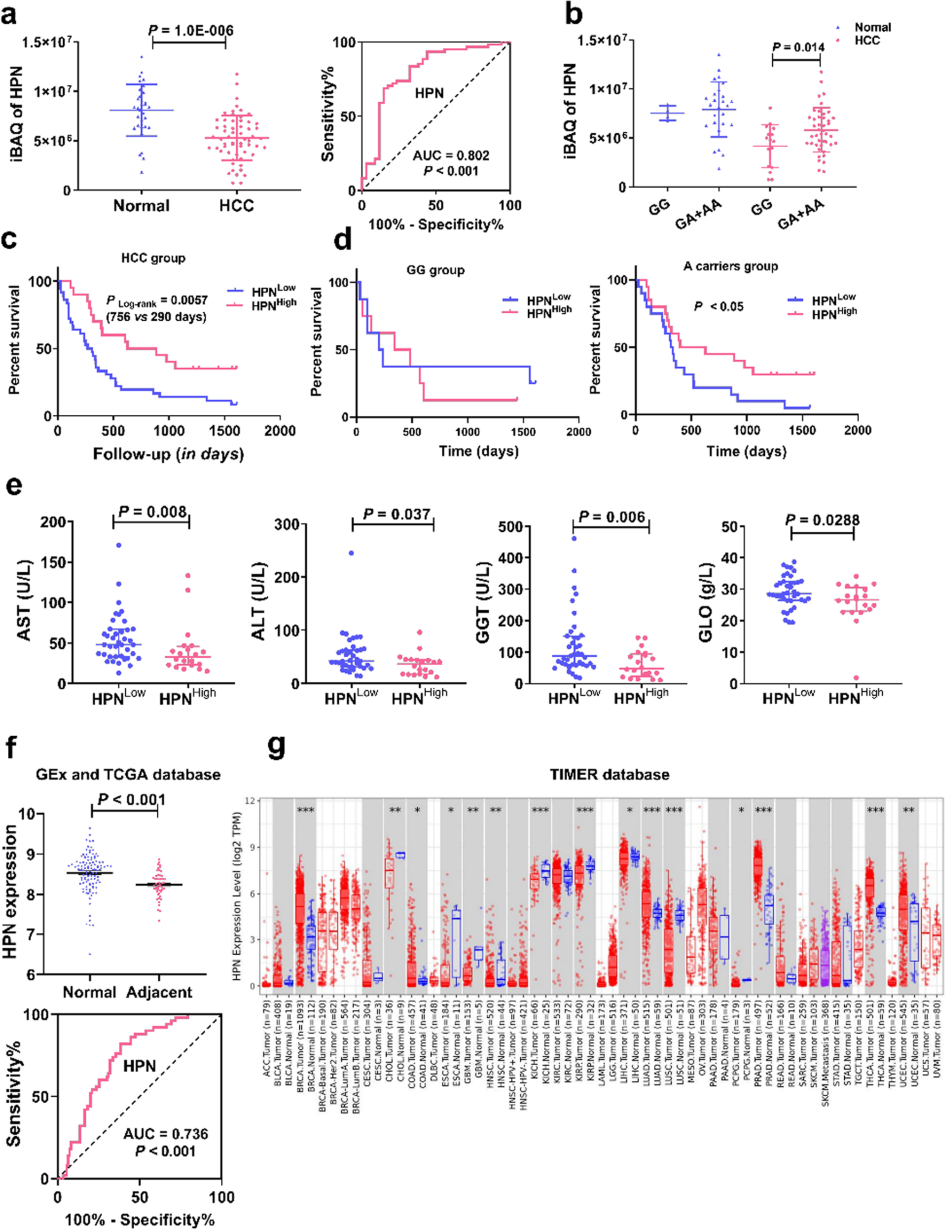
Significantly decreased HPN expression in HCC and associated with worse prognosis. **a** Decreased protein abundance of HPN in HCC patients and an ROC curve of HPN for diagnosis of HCC in the discovery cohort. **b** Increased protein abundance of HPN with *rs10954732* *GA* + *AA* genotypes in HCC patients. **c** Low expression of HPN conferring a poor prognosis in overall HCC patients in the discovery cohort. **d** Significantly altered OS between HPN high-expressing and low-expressing groups only in A allele carriers. **e** Patients with higher HPN levels were more likely to have lower AST, ALT, GGT and GLO. **f** Differential expression and ROC curve of HPN in adjacent tissues in HCC patients in TCGA database by comparison with normal subjects in the GTEx database. **g** Levels of HPN expression in tumor tissues of different tumor types from the TCGA database in TIMER. Note: *P < 0.05, **P < 0.01, ***P < 0.001. *HCC* hepatocellular carcinoma, *AST* transaminase, *ALT* alanine aminotransferase, *GGT* γ-glutamyl transpeptadase, *GLO* globulin

Hepsin was the only DEP with an AUC > 0.8 (Fig. 4a). In contrast to the significant down-regulation in the HCC group, expression of HPN was higher in the A allele carriers compared with wild-type (*P* = 0.014). Moreover, there was no significant difference in HPN expression when grouped by *G* > *A* mutation in normal subjects (Fig. 4b). That’s to say, lowered HPN expression was detected in HCC patients, while *A* allele carriers were more likely to have higher expression of HPN, findings further validating that the decreased HCC susceptibility underlying the *rs10954732* *A* allele might be related to increased HPN expression.

To further elucidate the prognostic value of HPN, a Kaplan–Meier survival analysis was performed with our discovery cohort. As shown in Fig. 4c, a lower level of HPN was associated with worse prognosis in HCC patients, evidenced by a prominently decreased (61.6%) OS in the HPN low-expressing group (290 *vs* 756 days, *P* = 0.0057). However, this association was only in patients genotyped with the A allele, and no difference in OS was found between HPN high- and low-expressing patients in the *GG* wild-type group (Fig. 4d). Similarly, patients with higher HPN expression were more likely to have lower levels of AST, ALT,γ-glutamyl transpeptidase (GGT) and globulin (GLO) (Fig. 4e), indicating that lower HPN expression is an indicator for poorer prognosis of HCC.

### Validation of lowered expression and the prognostic significance of HPN

Altogether, results from the discovery cohort strongly indicate that lower expression of HPN in A allele carriers is a risk factor for HCC occurrence and is correlated with poorer prognosis.

Lowered expression of HPN in HCC peritumoral tissues as compared with normal tissues was first confirmed by the validation set obtained from the GEx and TCGA databases (Fig. 4f), with a AUC at 0.736. Next, the analysis of RNA-seq data from the TIMER database also showed deregulated expression in tumor tissues from various cancers, including significantly lower expression in HCC (Fig. 4g). The lower expression of HPN in peritumor tissues and tumor tissues of HCC patients indicate a protective role for HPN in HCC occurrence.

**Fig. 5:**
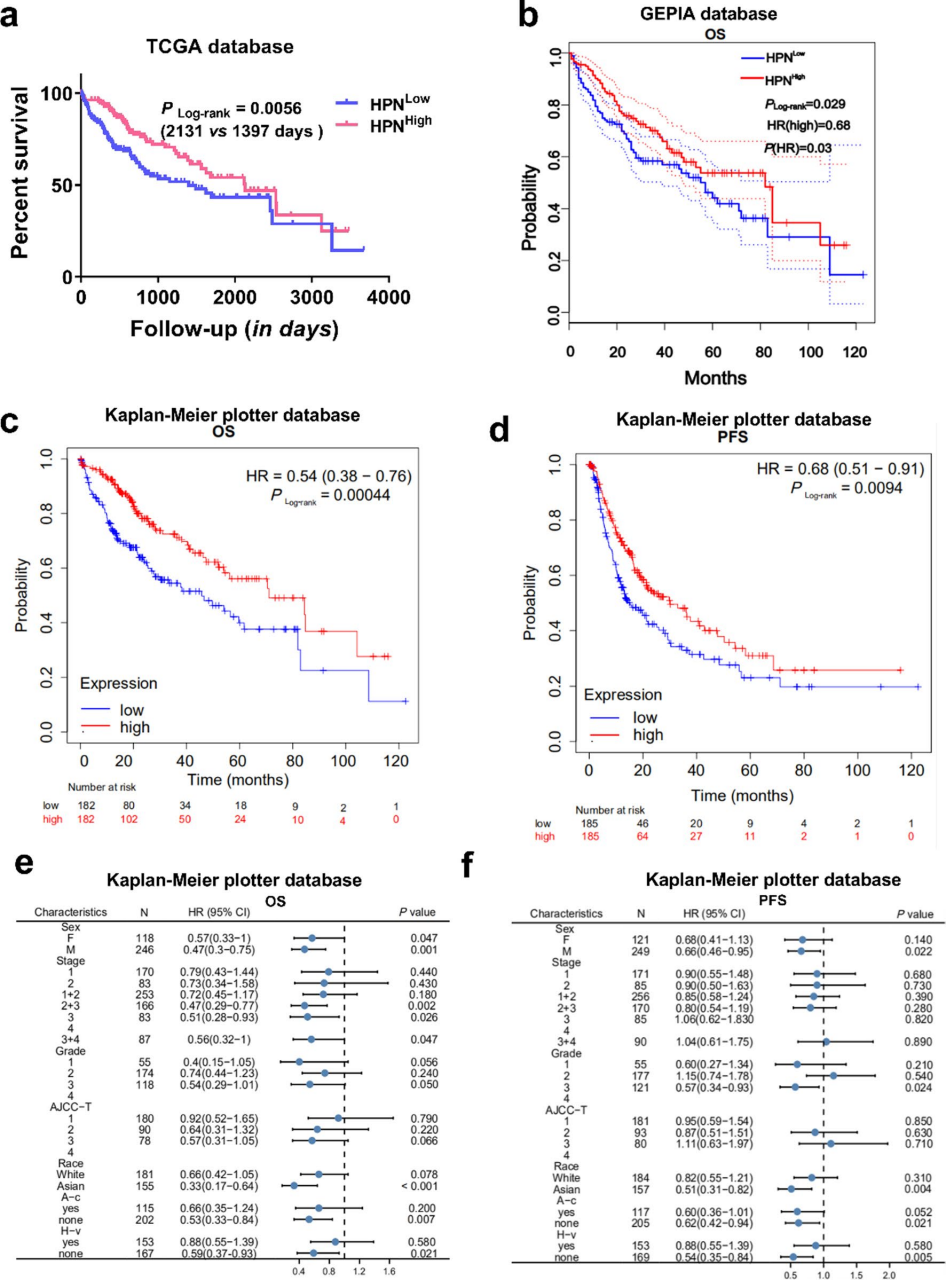
Prognostic value of highly-expressed and lowly-expressed HPN in HCC patients. **a**, **b** Low HPN expression was correlated with worse OS in the TCGA database (**a**) and the GEPIA database (**b**). Survival curves of OS (**c**) and PFS (**d**) in HCC using the Kaplan–Meier plot database (n = 364, n = 370). Correlation of HPN mRNA expression and OS (**e**) and PFS (**f**) in HCC with different clinicopathological factors by Kaplan–Meier plot (n = 364, n = 370). *OS* overall survival, *PFS* progression-free survival

The prognostic value of lower HPN expression was substantiated by a remarkably shortened OS of HCC patients (by 34.5%) from the TCGA database (1397 vs 2131 days, *P* = 0.0056) (Fig. 5a) and poor prognosis from the GEPIA database (HR = 0.68, *P* = 0.03) (Fig. 5b). Furthermore, this finding was again corroborated by analysis of the Kaplan–Meier plotter database. Low HPN expression was associated with worse prognosis in HCC (OS: HR = 0.54, 95% CI = 0.38 to 0.76, *P* = 0.00044; PFS: HR = 0.68, 95% CI = 0.51 to 0.91, *P* = 0.0094; Fig. 5c, d).

Subsequently, association between HPN expression and prognosis of HCC patients with different clinical characteristics was investigated. Notably, the expression of HPN was not always associated with prognosis of HCC. Specifically, a low HPN level correlated well with both worse OS (Fig. 5e) and PFS (Fig. 5f) in males (OS: HR = 0.47, *P* = 0.001; PFS: HR = 0.66, *P* = 0.022), Asians (OS: HR = 0.33, *P* < 0.001; PFS: HR = 0.51, *P* = 0.002) and patients without alcohol use (OS: HR = 0.53, *P* = 0.007; PFS: HR = 0.621, *P* = 0.021), without hepatitis viral infection (OS: HR = 0.59, P = 0.021; PFS: HR = 0.54, P = 0.005). In other cases, low expression of HPN was weakly or uncorrelated with poor prognosis, suggesting that the prognostic value of HPN expression was influenced by the clinical characteristics of HCC patients.

### Expression and prognostic value of HPN based on immune infiltration

**Fig. 6:**
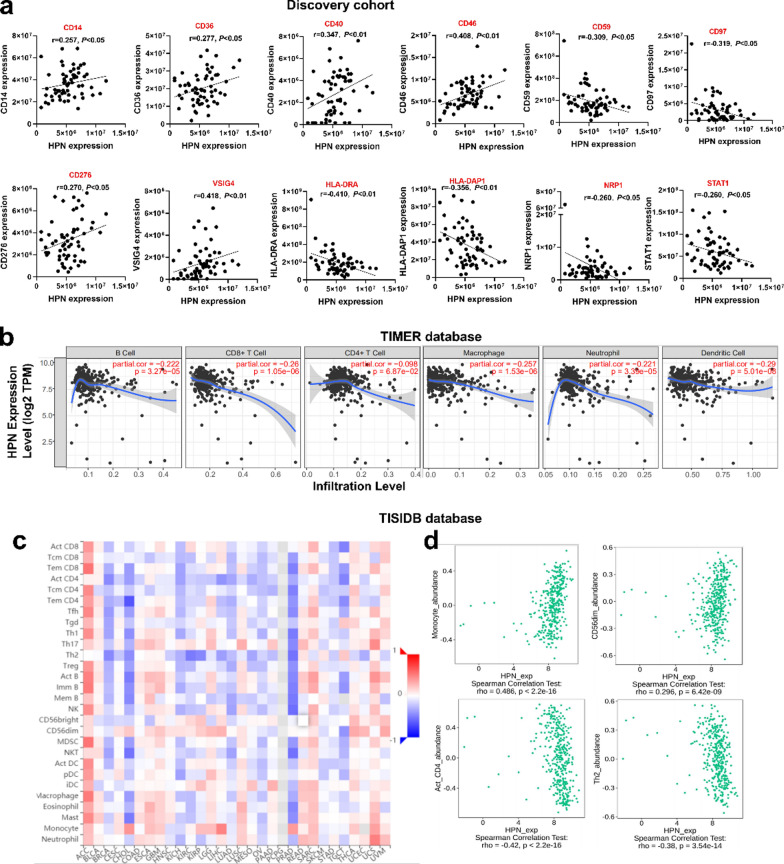
Correlation analysis of HPN expression and infiltration levels of immune cells in HCC tissues. **a** Correlation analysis of HPN abundance and expression of markers of immune cells based on proteomic analysis in the discovery cohort (n = 60). **b** Correlation between HPN expression and infiltration levels of immune cells in HCC by the TIMER database (n = 371).** c** Correlation between HPN level and twenty-eight tumor immune infiltrating cell subtypes among human heterogeneous cancers. **d** Top four immune cell subtypes with the greatest Spearman’s correlation value for HPN expression in HCC. *HCC* hepatocellular carcinoma

Since the important role of HPN in the immune system and the quantity and activity status of tumor-infiltrating lymphocytes can influence the prognosis of HCC patients, the correlation of HPN expression with markers of different subsets of immune cells in our data set was next analyzed. Our results revealed that HPN abundance correlated positively with immune cell gene marker levels, such as CD14, CD36, CD40, CD46, CD276 and VSIG4 and correlated negatively with the markers CD59, HLA-DRA, HLA-DAP1, NRP1 and STAT1 (Fig. 6a). These significant correlations between HPN expression and marker genes from immune cells like tumor-infiltrating monocytes, M2 macrophages, dendritic cells and T-helper cells suggests that HPN in A allele-related HCC might be involved in the regulation of tumor immune infiltration in HCC.

Consequently, this assumption was further tested by investigating the correlation between HPN expression and tumor-infiltrating immune cell subtypes in HCC patients in the TIMER database. We determined that the HPN level in tumor tissues was negatively associated with the infiltration levels of B cells (r = − 0.222, *P* = 3.27e−05), CD8 + T cells (r = − 0.26, *P* = 1.05e-06), macrophages (r = − 0.257, *P* = 1.53e−06), neutrophils (r = − 0.221, *P* = 3.39e-05) and dendritic cells (r = − 0.29, *P* = 5.01e-08) (Fig. 6b). No significant association was observed with CD4 + T cells. These results strongly suggest that HPN can modulate the infiltration of immune cells into tumor tissues in HCC.

To further support this inference, correlation between HPN expression in HCC and the levels of immune marker gene expression, which can represent the status of tumor-infiltrating immune cells, was assessed by analysis of the TIMER and GEPIA databases. The results indicate that HPN expression was in strongly negative correlation with 35 marker genes of infiltrating immune cells regardless of whether the correlation analysis was adjusted for purity or not (Additional file 1: Table S3), such as tumor-associated macrophages (TAM), M2 macrophages, monocytes, and neutrophils. Moreover, to further explore the regulation of immune molecules by HPN, correlation between HPN expression and 28 tumor-infiltrating lymphocytes (TILs) was analyzed across human heterogeneous cancers in the TISIDB database. The results show that HPN is associated with 13 TILs in HCC (Fig. 6c). Especially, monocytes (r = 0.486, *P* < 2.2e−16) and CD56dim natural killer cells (CD56dim) (r = 0.296, *P* = 6.42e−09) displayed the greatest positive correlations, while activated CD4 T cells (Act-CD4) (r = − 0.42, *P* < 2.2e−16) and Type 2T helper cells (Th2) (r = − 0.38, *P* = 3.54e−14) displayed the greatest negative correlations. These findings provide a potential mechanism underlying the oncogenic mechanisms of HPN, which might be by regulating the tumor microenvironment through tumor immunity and recruiting and regulating immune cells in HCC.

**Fig. 7:**
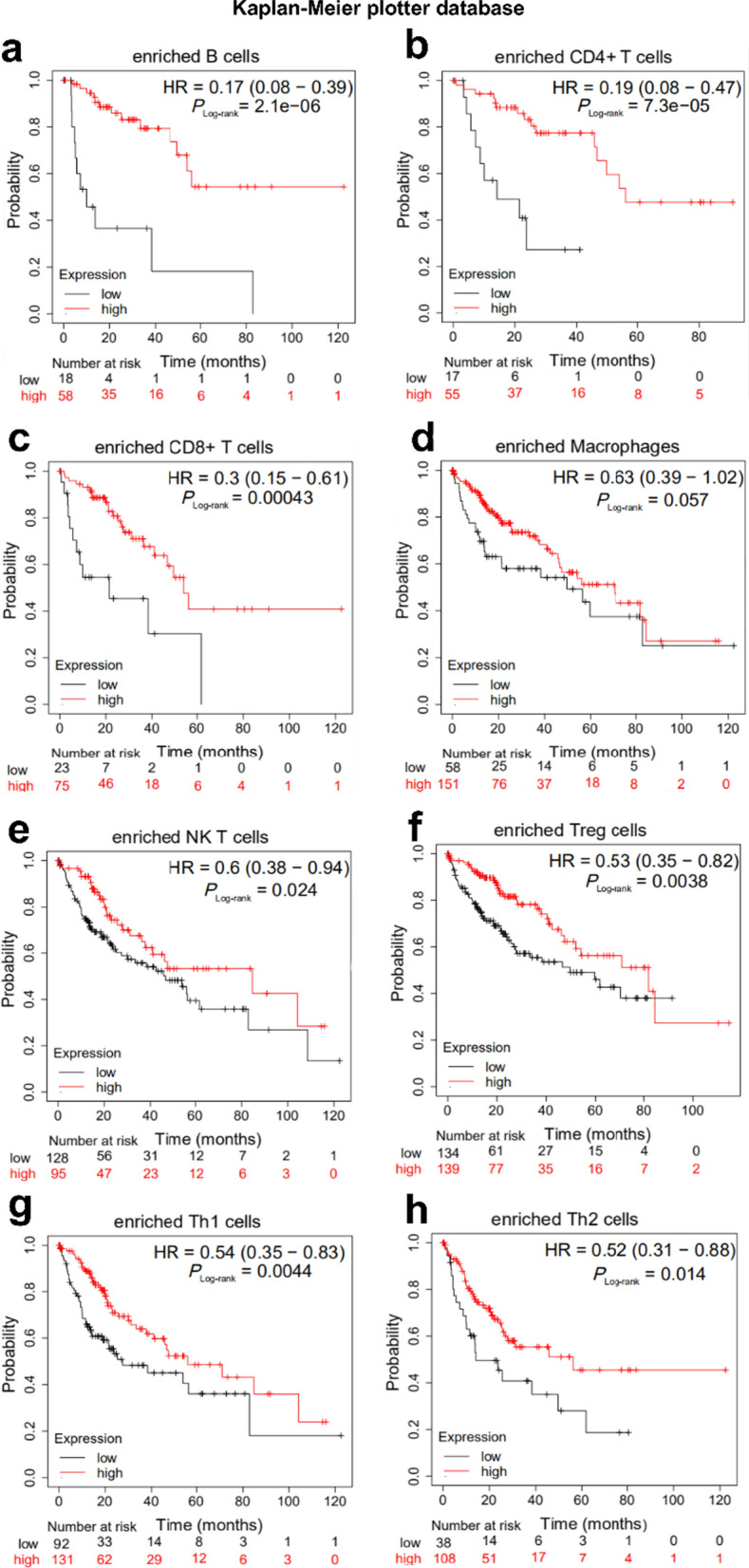
Kaplan–Meier survival curves between HPN high- and low-expressing groups in HCC based on immune cell subgroups. Low HPN level in enriched B cells (**a**), CD4 + memory T cells (**b**), CD8 + T cells (**c**), NK T cells (**e**), Treg cells (**f**), Th1 cells (**g**) and Th2 cells (**h**) is correlated with worse OS in HCC. For the enriched macrophages, low HPN level showed a decreasing trend in OS, though it did not reach statistical significance. *HCC* hepatocellular carcinoma

Kaplan–Meier plot analyses of HPN expression in HCC in B cells, CD4 + memory T cells, CD8 + T cells, macrophages, NK T cells, Treg T cells, Th1 cells, Th2 cells was next performed to assess the predictive potential of HPN in HCC based on immune cells. We found that HCC patients with lower HPN levels in enriched B cells (*P* = 2.1e−006), CD4 + memory T cells (*P* = 8.8e−03), CD8 + T cells (*P* = 8e−03), natural killer T cells (p = 5.8e−03), Treg T cells (p = 3.5e−03), type 1 T helper cells (*P* = 4.7e−03), type 2 T-helper cells (*P* = 3.5e−03) cohort were more likely to have a worse prognosis (Fig. 7a–h). A similar trend was found in macrophages, though it was not statistically significant. The above analysis suggests that low expression of HPN affects the prognosis of HCC patients through immune infiltration.

**Fig. 8:**
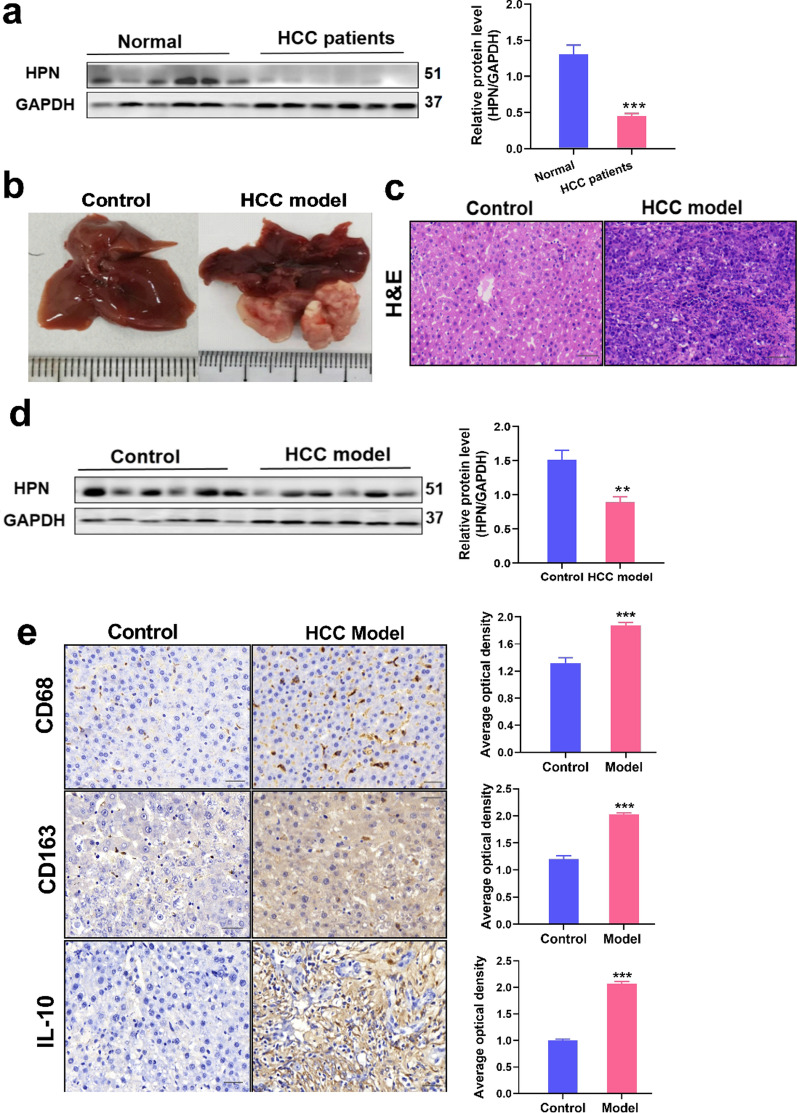
Verification of decreased HPN expression in HCC and correlation with expression of marker genes for immune cells. **a** Western blot analysis of HPN in another cohort of human samples. General picture (**b**), representative images of H&E staining (**c**) and western blot analysis of HPN (**d**) in a BALB/c mouse model. **e** Representative images of immunohistochemical staining for CD68, CD163 and IL-10 in a BALB/c mouse model (Scale bar, 100 μm). Data are expressed as mean ± SEM. Statistical analysis was performed according to a Mann–Whitney test. Normal means healthy subjects based on human liver samples. Control means control group based on the HCC mouse model. H&E, haematoxylin and eosin. **P < 0.01, ***P < 0.001 vs Normal or Control. *HCC* hepatocellular carcinoma. (n = 6 for each group)

Finally, to validate the proteomic results and database surveys, expression of HPN was measured by a WB method in another set of human liver samples from HCC patients and in a mouse HCC model. In agreement with proteomic findings, HPN levels were dramatically lower in peritumoral specimens of HCC patients than in normal livers (*P* < 0.001, Fig. 8a). Consistently, significantly lower HPN expression also was detected in a mouse HCC model (*P* < 0.01, Fig. 8b–d). The results further verified that lower HPN expression conferred increased HCC occurrence. Furthermore, expression of marker of tumor-infiltrating immune cells like CD68, CD163 and IL-10 significantly increased in a mouse HCC model group by paraffin specimen analysis (*P* < 0.001, Fig. 8e), which further demonstrates HPN-related tumorigenesis and progression related to tumor immune cell infiltration in HCC.

## Discussion

This study reports the association of the *POR* *rs10459732* (*G* > *A*) polymorphism with decreased susceptibility to HCC and better prognosis of HCC patients, which might be related to decreased CYP2E1 activity, and further with altered TME, mainly involved in immune responses. We further demonstrate that significantly down-regulated HPN expression is related to HCC risk and is a potential independent prognostic biomarker for HCC, which is strongly associated with clinicopathological features, poor prognosis, and infiltration status of immune cells both in our discovery cohort and in database surveys. Our study provides a new potential mechanism by which HPN may play an important role in A allele carriers for HCC susceptibility and prognosis through tumor immune infiltration.

Although there have been extensive studies on various genetic variations associated with either susceptibility or progression of HCC [33], few studies have been performed regarding POR polymorphisms, and with limited polymorphic sites. To date, only *POR A503V* has been confirmed as an HCC susceptibility polymorphism [20]. Here we report the novel finding that the *POR rs10459732* (*G* > *A*) polymorphism is correlated with decreased susceptibility to HCC and prognosis. Decreased CYP2E1 activity associated with *POR rs10459732 A* carriers can at least in part explain the decreased HCC susceptibility, which might be related to the critical role POR plays as an obligate electron donor for CYP enzymes [34]. This affects the activities of CYPs including CYP2E1, and the important role of CYP2E1 in the activation of procarcinogens and protoxins. This speculation is supported by previous reports from other groups [9, 35–39] as well as ours [16, 17, 40] that increased CYP2E1 activity is closely associated with oxidative stress, inflammation, and inflammation-related diseases like nonalcoholic steatohepatitis (NASH), hepatofibrosis, HCC, ovarian cancer, and so on.

High-resolution mass spectrometry (MS) was utilized here for proteome analysis of complex protein samples to uncover the potential mechanism underlying the* rs10459732* (*G* > *A*) polymorphism related to HCC susceptibility. We identified a significantly altered TME, mainly in immune responses, followed by inflammatory responses, which may be related to reduced HCC risk and better prognosis in the A allele carriers. Among the altered TME, HPN, which has been associated with the growth and progression of various cancers [25–28], might partly account for the A allele carriers association with HCC susceptibility and prognosis. Moreover, in line with our assumptions, further genotyping in validated cohorts in six HCC patients showed that four samples beared the *A* gene had higher expression trend of HPN compared with the two wild type *GG*.

Further database surveys and validated cohorts in HCC patients and an HCC mouse model (Fig. 8a, b) corroborated the down-regulation of HPN in HCC. It seems paradoxical that HPN plays a role in HCC based on its down-regulation in our and other reports [41, 42], as its level was up-regulated in other cancers such as ovarian carcinoma, renal cell carcinoma, and prostate cancer as reported in previous studies [43–45]. Thus, the effect of HPN on carcinogenesis might be tissue- and cell type-specific, which may explain the conflicting literature on the influence of HPN in different cancer cells.

TME, consisting of tumor cells, stromal cells, extracellular matrix and infiltrating immune cells, is known to be highly immunosuppressive for HCC [46]. Tumor-infiltrating lymphocytes play vital roles in tumor development. HPN, a protein involved in immune reactions and metabolism, was previously reported to regulate the TME [26, 28, 47–50]. Here, we report that HPN expression is associated with several immune infiltrating cells and many immune cell makers, including T helper cells (Th1, Th2 and Tfh), in HCC through correlation analysis. These connections may indicate underlying mechanisms for HPN regulation of T cell function in HCC. Therefore, it may be related to the poor prognosis of HCC by recruiting and regulating immune cells, which was validated by lower expression of HPN in a variety of immune cells in a cohort of HCC showing a worse prognosis from the Kaplan Meier-Plot database analysis (Fig. 7). We speculate that lowered HPN expression, along with tumor associated macrophages (TAMs), regulatory T cells (Tregs), and myeloid-derived suppressor cells (MDSCs) as well as tumor-associated dendritic cells (tDCs) together suppress anti-tumor immune responses, and lead to tumor-mediated immune escape, constituting the immunosuppressive TME [51].

In summary, our study suggests that *POR rs10954732* (*G* > *A*) is significantly associated with decreased HCC risk and prolonged OS in HCC patients, with the underlying mechanism being down-regulated HPN expression. The lower HPN level is a potential independent prognostic biomarker for HCC, and is strongly associated with clinicopathological features, poor prognosis, and immune cell infiltration. Furthermore, our study offers insights for further studies on tumor immunotherapy based on the potential that HPN may affect the prognosis of HCC through tumor immune infiltration. Relatively low levels of HPN in HCC may indicate greater risk of tumor relapse after treatment and close medical supervision will be necessary for such patients. The current study is a preliminary part of a larger study, validation of the *POR* polymorphism-related HCC risk and prognosis in additional larger HCC cohorts, and the exact detailed mechanism by which HPN regulates tumor infiltration of immune cells in HCC are needed to verify our results.

## Supplementary Information

**Additional file 1**:** Table S1**. Basic demographic and clinical parameters characteristics of human liver tissue samples.** Table S2**. Hardy-Weinberg test for SNP loci of POR.** Table S3**. Correlation analysis between HPN and relate genes and markers of immune cells in TIMER

## Data Availability

The datasets generated during and/or analysed during the current study are not publicly available due (REASON WHY DATA ARE NOT PUBLIC) but are available from the corresponding author on reasonable request.
