# Supplementary material for: The POR rs10954732 polymorphism decreases susceptibility to hepatocellular carcinoma and hepsin as a prognostic biomarker correlated with immune infiltration based on proteomics
Source: J Transl Med. 2022 Feb 14;20:88. doi: 10.1186/s12967-022-03282-1 (PMC8842912; doi:10.1186/s12967-022-03282-1)
Supplement: Supplementary file 1 — Additional file 1: Table S1. Basic demographic and clinical parameters characteristics of human liver tissue samples. Table S2. Hardy-Weinberg test for SNP loci of POR. Table S3. Correlation analysis between HPN and relate genes and markers of immune cells in TIMER [file 12967_2022_3282_MOESM1_ESM.docx]

**Supplementary Table 1 Basic demographic and clinical parameters characteristics of human liver tissue samples**

| Characteristics | Normal (n= 85) | HCC (n = 100) | *P* value |
| --- | --- | --- | --- |
| Gender |  |  |  |
| Male, n (%) | 27 (31.8) | 84 (84.0) |  |
| Female, n (%) | 58 (68.2) | 16 (16.0) | 5.92×10^-13^ |
| Smoking |  |  |  |
| Yes, n (%) | 15 (17.6) | 50 (50.0) |  |
| No, n (%) | 70 (82.4) | 50(50.0) | 4.36×10^-6^ |
| Drinking |  |  |  |
| Yes, n (%) | 16 (18.8) | 37 (37.0) |  |
| No, n (%) | 69 (81.2) | 63 (63.0) | 0.006 |
| Age (years) | 48 (43~54) | 54 (46~62) | 0.001 |
| ALT (U/L) | 19.0 (13.5~26.5) | 33.5 (21.0~45.0) | 1.57×10^-8^ |
| AST (U/L) | 19.0 (15.0~23.0) | 36.5 (27.3~48.0) | 1.00×10^-13^ |
| GGT (U/L) | 26.0 (16.0~52.3) | 67.5 (42.0~121.0) | 2.17×10^-10^ |
| TP (g/L) | 67.8 (63.4~73.3) | 70.3 (67.0~72.7) | 0.028 |
| GLO (g/L) | 26.5 (23.7~29.4) | 28.2 (25.4~30.9) | 0.007 |
| ALP (U/L) | 72.5 (58.8~87.3) | 103.0 (80.5~130.8) | 5.00×10^-9^ |
| PT(s) | 11.7(11.0~12.4) | 12.6(12.1~13.6) | 2.98×10^-8^ |

HCC, hepatocellular carcinoma; ALT, alanine aminotransferase; AST, aspartate aminotransferase; GGT, gamma-glutamyl transferase; TP, total protein; GLO, globulin; ALP, alkaline phosphatase; PT, plasma prothrombin time. For age, ALT, AST, GGT, TP, GLO, ALP and PT, data was expressed as median (25th­~75th).

**Supplementary Table 2 Hardy-Weinberg test for SNP loci of *POR***

| SNP loci | Normal | |  | HCC | |
| --- | --- | --- | --- | --- | --- |
|  | χ2 | *P* value | | χ2 | *P* value |
| rs10954732 | 2.055 | 0.152 | | 1.130 | 0.288 |
| rs2286822 | 0.337 | 0.562 | | 0.011 | 0.918 |
| rs1135612 | 0.107 | 0.744 | | 1.220 | 0.269 |
| rs1057868 | 0.048 | 0.827 | | 0.504 | 0.478 |

HCC, hepatocellular carcinoma; SNP, single-nucleotide polymorphism.

**Supplementary Table 3 Correlation analysis between HPN and relate genes and markers of immune cells in TIMER.**

| Description | Gene marker | HCC | | | |  | CHOL | | | |
| --- | --- | --- | --- | --- | --- | --- | --- | --- | --- | --- |
|  |  | None | | Purity | |  | None | | Purity | |
|  |  | Core | *P* | Core | *P* |  | Core | *P* | Core | *P* |
| CD8+T cell | CD8A | -0.170 | ** | -0.156 | ** |  | -0.467 | ** | -0.386 | * |
|  | CD8B | -0.120 | ** | -0.145 | ** |  | -0.567 | *** | -0.515 | ** |
| T cell (general) | CD3D | -0.178 | *** | -0.167 | ** |  | -0.371 | * | -0.258 | 0.134 |
|  | CD3E | -0.141 | ** | -0.133 | * |  | -0.473 | ** | -0.377 | * |
|  | CD2 | -0.148 | ** | -0.143 | ** |  | -0.454 | ** | -0.354 | * |
| T cell exhaustion | CTLA4 | -0.281 | *** | -0.279 | *** |  | -0.569 | *** | -0.52 | ** |
|  | LAG3 | -0.188 | *** | -0.189 | *** |  | -0.333 | * | -0.246 | 0.155 |
|  | HAVCR2 | -0.241 | *** | -0.251 | *** |  | -0.477 | ** | -0.395 | * |
|  | GZMB | -0.195 | *** | -0.191 | *** |  | -0.383 | * | -0.288 | 0.093 |
|  | PDCD1 | -0.167 | ** | -0.146 | ** |  | -0.162 | 0.345 | -0.084 | 0.633 |
| B cell | CD19 | -0.167 | ** | -0.156 | ** |  | -0.418 | * | -0.317 | 0.063 |
|  | CD79A | -0.171 | *** | -0.168 | ** |  | -0.497 | ** | -0.416 | * |
| Monocyte | CD86 | -0.280 | *** | -0.301 | *** |  | -0.497 | ** | -0.412 | * |
|  | CSF1R | -0.217 | *** | -0.235 | *** |  | -0.231 | 0.175 | -0.12 | 0.491 |
| TAM | CCL2 | -0.141 | ** | -0.131 | * |  | 0.028 | 0.870 | 0.11 | 0.531 |
|  | CD68 | -0.213 | *** | -0.203 | *** |  | -0.324 | 0.055 | -0.259 | 0.134 |
|  | IL10 | -0.193 | *** | -0.188 | *** |  | -0.363 | * | -0.218 | 0.209 |
| M1 Macrophage | NOS2 | -0.008 | 0.885 | -0.025 | 0.641 |  | 0.219 | 0.200 | 0.233 | 0.179 |
|  | IRF5 | -0.118 | * | -0.127 | * |  | -0.205 | 0.229 | -0.141 | 0.418 |
|  | PTGS2 | -0.066 | 0.201 | 0.058 | 0.279 |  | -0.471 | ** | -0.403 | * |
| M2 Macrophage | CD163 | -0.150 | ** | -0.147 | ** |  | -0.425 | * | -0.328 | * |
|  | VSIG4 | -0.122 | * | -0.113 | * |  | -0.332 | * | -0.227 | 0.189 |
|  | MS4A4A | -0.154 | ** | -0.154 | ** |  | -0.434 | ** | -0.323 | 0.058 |
| Neturophile | CEACAM8 | -0.056 | 0.286 | -0.046 | 0.396 |  | -0.204 | 0.233 | -0.207 | 0.233 |
|  | ITGAM | -0.252 | *** | -0.263 | *** |  | -0.242 | 0.154 | -0.179 | 0.304 |
|  | CCR7 | -0.092 | 0.075 | -0.079 | -0.141 |  | -0.499 | ** | -0.412 | * |
| Natural killer cell | KIR2DL1 | 0.006 | 0.908 | 0.009 | 0.873 |  | -0.103 | 0.552 | -0.061 | 0.729 |
|  | KIR2DL3 | -0.171 | *** | -0.182 | *** |  | -0.189 | 0.270 | -0.161 | 0.355 |
|  | KIR2DL4 | -0.117 | * | -0.104 | 0.054 |  | -0.197 | 0.249 | -0.137 | 0.432 |
|  | KIR3DL1 | -0.084 | 0.106 | -0.079 | 0.141 |  | -0.241 | 0.157 | -0.208 | 0.230 |
|  | KIR3DL2 | -0.082 | 0.114 | -0.081 | 0.132 |  | -0.171 | 0.319 | -0.17 | 0.328 |
|  | KIR3DL3 | 0.029 | 0.571 | 0.053 | 0.324 |  | -0.007 | 0.966 | 0.037 | 0.831 |
|  | KIR2DS4 | -0.003 | 0.951 | -0.016 | 0.770 |  | -0.427 | ** | -0.405 | * |
| Dendritic cell | HLA-DPB1 | -0.200 | *** | -0.21 | *** |  | -0.333 | * | -0.227 | 0.190 |
|  | HLA-DQB1 | -0.158 | ** | -0.159 | ** |  | -0.287 | 0.090 | -0.217 | 0.210 |
|  | HLA-DRA | -0.223 | *** | -0.23 | *** |  | -0.368 | * | -0.260 | 0.131 |
|  | HLA-DPA1 | -0.177 | *** | -0.178 | *** |  | -0.395 | * | -0.293 | 0.087 |
|  | ITGAX | -0.232 | *** | -0.245 | *** |  | -0.494 | ** | -0.410 | * |
|  | NRP1 | -0.237 | *** | -0.222 | *** |  | -0.219 | 0.199 | -0.129 | 0.459 |
|  | CD1C | -0.101 | 0.052 | -0.102 | 0.057 |  | -0.282 | 0.095 | -0.168 | 0.334 |
| Th1 | TBX21 | -0.146 | ** | -0.154 | ** |  | -0.33 | * | -0.188 | 0.278 |
|  | STAT4 | -0.026 | 0.613 | -0.015 | 0.778 |  | -0.18 | 0.291 | -0.094 | 0.590 |
|  | STAT1 | 0.178 | *** | -0.18 | *** |  | 0.153 | 0.370 | 0.220 | 0.204 |
| Th2 | GATA3 | -0.161 | ** | -0.159 | ** |  | -0.344 | * | -0.216 | 0.213 |
|  | STAT6 | -0.003 | 0.960 | 0.014 | 0.779 |  | 0.103 | 0.549 | 0.097 | 0.579 |
|  | STAT5A | -0.204 | *** | -0.189 | *** |  | -0.344 | * | -0.305 | 0.075 |
|  | IL13 | -0.014 | 0.791 | -0.011 | 0.84 |  | 0.11 | 0.524 | 0.203 | 0.241 |
| Tfh | BCL6 | -0.15 | ** | -0.144 | ** |  | -0.369 | * | -0.362 | * |
|  | IL21 | -0.063 | 0.224 | -0.078 | 0.146 |  | -0.024 | 0.891 | 0.069 | 0.694 |

**P*<0.01; ***P*<0.001; ****P*<0.0001
